# Platelets and Infection – An Emerging Role of Platelets in Viral Infection

**DOI:** 10.3389/fimmu.2014.00649

**Published:** 2014-12-18

**Authors:** Alice Assinger

**Affiliations:** ^1^Department of Physiology and Pharmacology, Medical University of Vienna, Vienna, Austria; ^2^Department of Medicine, Center for Molecular Medicine, Karolinska University Hospital, Stockholm, Sweden

**Keywords:** platelets, viruses, thrombocytopenia, thrombosis, immune response

## Abstract

Platelets are anucleate blood cells that play a crucial role in the maintenance of hemostasis. While platelet activation and elevated platelet counts (thrombocytosis) are associated with increased risk of thrombotic complications, low platelet counts (thrombocytopenia) and several platelet function disorders increase the risk of bleeding. Over the last years, more and more evidence has emerged that platelets and their activation state can also modulate innate and adaptive immune responses and low platelet counts have been identified as a surrogate marker for poor prognosis in septic patients. Viral infections often coincide with platelet activation. Host inflammatory responses result in the release of platelet activating mediators and a pro-oxidative and pro-coagulant environment, which favors platelet activation. However, viruses can also directly interact with platelets and megakaryocytes and modulate their function. Furthermore, platelets can be activated by viral antigen–antibody complexes and in response to some viruses B-lymphocytes also generate anti-platelet antibodies. All these processes contributing to platelet activation result in increased platelet consumption and removal and often lead to thrombocytopenia, which is frequently observed during viral infection. However, virus-induced platelet activation does not only modulate platelet count but also shape immune responses. Platelets and their released products have been reported to directly and indirectly suppress infection and to support virus persistence in response to certain viruses, making platelets a double-edged sword during viral infections. This review aims to summarize the current knowledge on platelet interaction with different types of viruses, the viral impact on platelet activation, and platelet-mediated modulations of innate and adaptive immune responses.

## Introduction

From an evolutionary perspective, the cellular mediators of hemostasis and immune defense have not always been separated. In invertebrates, a cell type called hemocyte protects the host from invading microbes and the same cell also prevents “blood” (i.e., hemolymph) loss upon injury by triggering coagulation (1, 2). In highly developed species, hemostasis and immune response have been divided and leukocytes resume the immune response functions, while platelets maintain hemostasis (2).

For decades, these two systems were thought to act independently, but this concept has recently been challenged. Emerging evidence suggests that the boundaries between coagulation and immune defense are actually not as clear-cut as originally supposed. A new concept of involvement of immune cells in hemostasis, termed immunothrombosis, has been recently proposed, which assumes a function of innate immune cells in thrombosis (3). On the other hand, an important role of platelets in immune response becomes more and more apparent (4–7).

In 1882, Bizzozero discovered platelets as the third morphological element in blood and elucidated the function of these cells in hemostasis and thrombosis (8). For a long time, platelets and their granule content were thought to mainly mediate the activation of the coagulation system and the recruitment of further platelets to stop blood loss upon injury. Unwanted platelet activation was noted to occur as a response to internal injuries, for example, denudation and erosion of the endothelial surface or rupture of an atherosclerotic plaque (9). Consequently, platelets were assumed to be responsible for the lethal steps of cardiovascular diseases, where unstable thrombi occlude small vessels, thereby compromising oxygen supply of target organs. Nevertheless, platelets are involved in far more processes and they respond to and interact with far more triggers than initially thought. Besides their central role in hemostasis, platelets assist and modulate inflammatory reactions and immune responses by direct interaction with leukocytes as well as endothelial cells and via release of soluble inflammatory mediators that enhance recruitment of leukocytes and trigger their activation (2, 4, 6, 10). Platelets also express surface receptors, such as lectins, integrins, and toll-like receptors (TLR), allowing them to directly interact with several pathogens. Moreover, they express Fc receptors by which they can recognize immunocomplexes.

Thus, platelet activation does not only occur in response to injury and is not only limited to hemostatic processes but also modulates host response and virus survival (3–5, 7, 10–12). Therefore, potential immune-modulatory side effects should be considered during anti-platelet therapy.

To date, the role of platelets in response to invading pathogens is not fully understood. Platelet–microbe interactions seem to be beneficial for the host due to up-regulation of immune responses. However, platelet interaction with invading pathogens is also speculated to benefit virus or bacteria, as platelets provide shelter against leukocytes, antiviral agents, and antibiotics. Moreover, the stickiness of platelets might attenuate microbe–endothelial interactions and facilitate infection by similar mechanisms to those recently described for platelet interactions with circulating tumor cells (13).

This review aims to summarize the current knowledge on platelet interaction with different types of viruses, the viral impact on platelet activation, and platelet-mediated modulations of innate and adaptive immune responses.

## Mechanisms of Virus-Induced Thrombocytopenia

**Figure 1:**
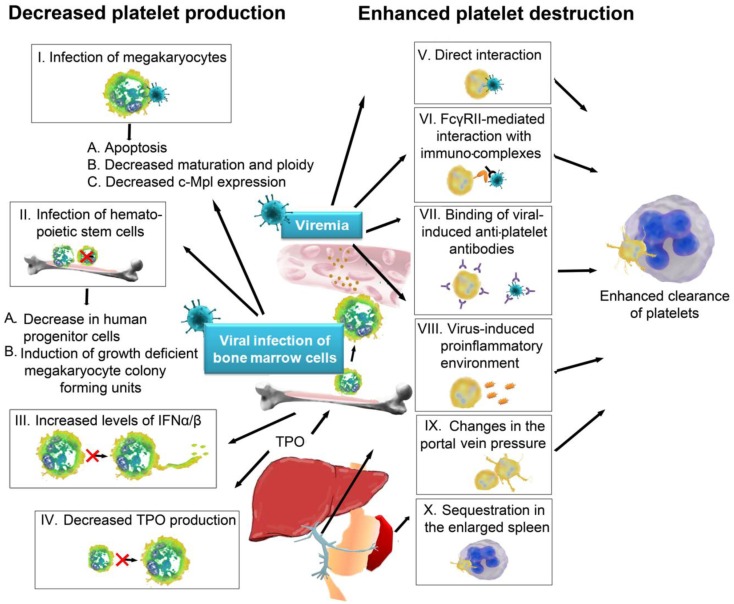
**Mechanisms of virus-induced thrombocytopenia: thrombocytopenia occurs either via decreased platelet production or increased platelet destruction**. Viruses can trigger a decrease in platelet production by (I) infection of megakaryocytes, which can lead to (A) apoptosis of megakaryocytes, (B) decreased maturation and ploidy of megakaryocytes, or (C) decreased expression of thrombopoietin receptor c-Mpl. Viruses can also infect hematopoietic stem cells (II), which results (A) in a decrease of progenitor cells and (B) the induction of growth deficient megakaryocyte colony forming units, due to disturbed cytokine production by the infected cells in the bone marrow. Viruses can further indirectly influence platelet production (III) via induction of IFNα/β, which suppresses proplatelet formation or (IV) by targeting and modulating liver functions, which are important for the production of megakaryocyte growth and development factor thrombopoietin. Another mechanism for how viruses cause thrombocytopenia is by favoring platelet destruction, which frequently occurs during viremia. Viruses can either (V) directly interact with platelets or (VI) platelets recognize immunocomplexes of IgGs and viral antigens. Antiviral antigens often show cross-reactivity with platelet surface integrins (VII), which provides another mechanism of virus-induced destruction of platelets. The virus-induced pro-inflammatory environment itself often leads to further platelet activation in viremic patients (VIII). Additionally, changes in the portal vein pressure (IX) and an enlarged spleen (X) can serve as trigger for platelet activation. Once activated, platelets are recognized by circulating leukocytes or by cells in spleen and liver and rapidly cleared from the circulation. IFN, interferon, TPO, thrombopoietin; FcγRII, Fc receptor γ R II.

**Figure 2:**
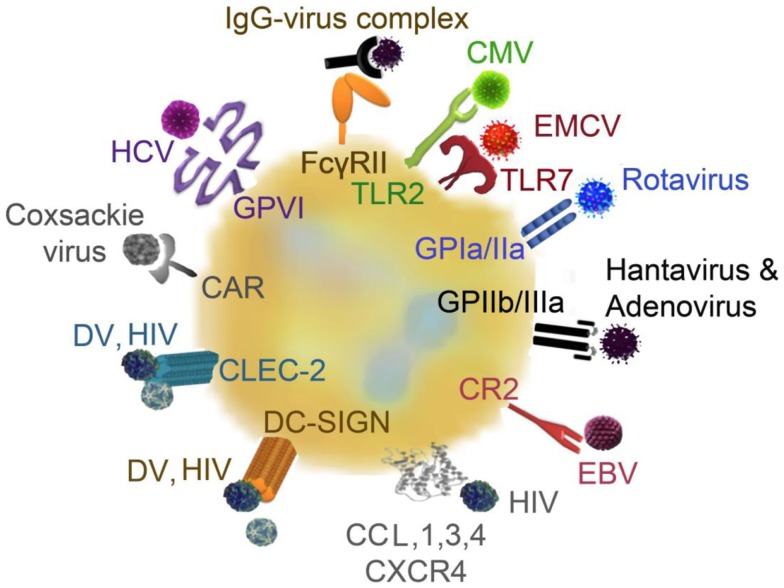
**Platelet receptors for viruses: platelets and viruses can directly interact via a plethora of surface receptors**. CMV binds to platelets via TLR2, EMCV interacts via TLR7, rotavirus utilizes GPIa/IIa to bind to platelets and hantavirus and adenovirus interact with platelets via GPIIb/IIIa. EBV–platelet interaction occurs via CR2. HIV and DV bind to lectin receptors such as CLEC-2 and DC-SIGN. HIV further interacts with CXCR4 and CCL3 and CCL5. Platelets express the Coxsackie virus-specific receptor, CAR, and HCV interacts with platelets via GPVI. CAR, Coxsackie-adenovirus receptor; CLEC-2, C-type lectin domain family 2; CCL, chemokine (C–C motif) ligand; CMV, cytomegalovirus; CR, complement receptor; CXCR4, C–X–C chemokine receptor type 4; EBV, Epstein–Barr virus; EMCV, encephalomyocarditis virus; DC-SIGN, dendritic cell-specific intercellular adhesion molecule-3-grapping non-integrin; DV, Dengue virus; FcγRII, Fc receptor γ II; GP, glycoprotein; HCV, hepatitis virus C; HIV, human immunodeficient virus; IgG, immunoglobulin G; TLR, toll-like receptor;

Platelets are, after erythrocytes, the second most abundant cell population in the blood. Normal platelet counts range from 150,000 to 450,000 platelets per microliter. Thrombocytopenia, which represents a drop in platelet count caused by either decreased platelet production or increased platelet destruction, is associated with an increased bleeding risk. Thrombocytopenia is frequent following viral infections and viruses use a variety of distinct strategies to decrease the levels of circulating platelets. The different mechanisms of how virus infection can interfere with platelet production and might trigger platelet destruction are summarized in the first chapter and Figure 1. As virus-mediated thrombocytopenia is often multifactorial and differs between virus infections, the mechanisms by which viruses trigger thrombocytopenia are separately discussed for the most prominent viruses and an overview on the receptors involved in direct platelet–virus interactions is depicted in Figure 2.

## Decreased Platelet Production

Platelets are produced in the bone marrow by megakaryocytes, which develop from hematopoietic stem cells. Megakaryocytes first undergo linage commitment, followed by endomitosis resulting in polyploidy (14). Polyploidy is a state where one cell comprises multiple sets of chromosomes and this represents an important step in megakaryopoiesis. After endoplasmatic maturation, megakaryocytes form proplatelets, which bud off thousands of platelets and microparticles into the blood stream (15). Megakaryopoiesis is triggered by a variety of cytokines (e.g., GM-CSF, IL-3, IL-6, IL-11, FGF4, and SDF-1), with thrombopoietin (TPO) being the most important. Nevertheless, TPO does not only trigger megakaryocyte development but also fulfils an important role in maintaining stem cells (16).

Viruses can modulate platelet production at various steps of development. They are able to influence the cytokine profile of the host, resulting in altered TPO production in the liver. Examples of this include: simian immunodeficiency virus (SIV), which triggers TPO production via up-regulation of tumor growth factor (TGF) β (17); human herpes virus 6, which can interfere with TPO-inducible megakaryocytic colony formation (18); human herpes virus 7, which impairs the survival and differentiation of megakaryocytes (19).

Some viruses also directly interfere with TPO production by destruction of liver tissue as shown for hepatitis C virus (HCV) (20). The resulting drop in TPO production results in delayed megakaryocyte development and a decrease in platelet production. Other viruses infect bone marrow stromal cells and hematopoietic stem cells, resulting in altered cytokine production and decreased number of progenitor cells, thereby disturbing hematopoiesis (21).

Human immunodeficiency virus (HIV), cytomegalovirus (CMV), and HCV are known to replicate in megakaryocytes (22–24) and many more viruses can interact with megakaryocytes modulating their proliferation and function (23, 25–29). Viral infection of megakaryocytes can increase apoptosis and decreases the maturation and ploidy of megakaryocytes. Moreover, virus-infected megakaryocytes have been shown to express less surface c-Mpl, which is the receptor for TPO (30). An overview on the different mechanisms on how viruses can interfere with platelet production is depicted in Figure 1.

## Enhanced Platelet Destruction

While thrombocytopenia induced by decreased platelet production is observed at later stages of infection, rapidly induced thrombocytopenia in response to viral infections is mediated via enhanced platelet destruction.

The most rapid way of platelet destruction occurs via direct interaction between platelets and viruses. Platelet–virus interaction can occur via a variety of receptors and are mainly mediated by integrins, surface lectins, and TLR (25, 31, 32) (see Figure 2). Platelets can bind CMV via TLR2, which triggers platelet activation and degranulation and results in enhanced platelet interaction with neutrophils (32). Encephalomyocarditis virus (EMCV) was recently shown to interact with platelet TLR7, which also leads to platelet degranulation and direct platelet–neutrophil interactions (33). Platelet fragments subsequently get phagocytized by neutrophils, thereby contributing to the drop in platelet count (33).

Rotavirus utilizes the collagen receptor GPIa/IIa to bind to platelets (25, 34), while hantavirus and adenoviruses interact with platelets via the fibrinogen receptor GPIIb/IIIa (35). However, GPIIb/IIIa does not seem to be the unique receptor for platelet–adenovirus interaction, as inhibition of GPIIb/IIIa does not alter platelet internalization of adenoviruses (36). In addition, hantavirus-infected endothelial cells can bind to quiescent platelets via GPIIb/IIIa (37), which results in platelet activation and clearance but might also influence vascular permeability (38).

GPIIb/IIIa is the most abundant platelet integrin and displays bi-directional signaling functions. Inside-out signaling, which is positively regulated by various platelet agonists, is mediated by intracellular protein–protein interactions and biochemical reactions that regulate GPIIb/IIIa affinity (39). These intracellular processes triggering GPIIb/IIIa activation are complex and include recruitment of talin, which separates the GPIIb and the GPIIIa subunits, and kindlins, which are involved in GPIIb/IIIa activation (40) independent of talin recruitment (41). Further, G protein subunit Gα_13_ directly binds to the cytoplasmic domain of GPIIIa and promotes ligand binding to GPIIb/IIIa (42).

Outside-in signaling via receptor binding promotes actin polymerization and platelet spreading (39) and can thereby enhance virus attachment to endothelial cells but also promote platelet clearance.

Epstein–Barr virus (EBV) interaction with platelets occurs via complement receptor 2 (CR2) (43). HIV and dengue virus activate platelets by binding to lectin receptors such as C-type lectin domain family 2 (CLEC-2) and cell-specific intercellular adhesion molecule-3-grapping non-integrin (DC-SIGN) (44). Platelets and/or megakaryocytes can further interact with HIV envelope proteins via C–X–C chemokine receptor type 4 (CXCR4) or via chemokine (C–C motif) ligand (CCL) 3 (MIP-1α) and 5 (RANTES) (25). However, HIV-1 changes its co-receptor usage from CCR5 to CXCR4 only after many years of infection and this receptor change represents a switch to non-CD4-dependent platelet activation at late stages of disease.

In addition, platelets express the Coxsackie virus-specific Coxsackie-adenovirus receptor (CAR) (45) and HCV interacts with platelets via collagen receptor GPVI (46).

These direct interactions often result in platelet activation and adhesion of activated platelets to leukocytes. Platelet binding to neutrophils triggers phagocytosis of platelets (33, 47) and platelet activation itself promotes platelet clearance in spleen and liver (48).

However, platelets are not only activated by direct interactions with viruses. Host defense mechanisms in response to viral infections can also lead to platelet activation. For example, many viral infections lead to systemic inflammation, which in turn triggers platelet activation and decreases platelet life span (49). Among others, influenza virus, rhinovirus, and CMV infection result in up-regulation of cytokines, such as interleukin 6 (IL-6), in target cells (50).

Platelets can be activated by these cytokines, leading to platelet–leukocyte interactions, which foster leukocyte and endothelial activation, further amplifying platelet activation and enhancing their clearance by splenic macrophages or Kupffer cells in the liver (45). Monocytes that encounter dengue virus, for example, start generating platelet activating factor (PAF) (51), which is a lipid mediator that triggers platelet activation. This leads to enhanced apoptosis of platelets and accelerates platelet clearance in secondary dengue infection (52).

Several virus infections activate the coagulation cascade via induction of tissue factor (TF) expression in target cells. Generation of thrombin by the activated coagulation cascade causes platelet activation and subsequent clearance via protease activating receptor (PAR) signaling (53). PARs on platelets, endothelial cells, and leukocytes are important modulators during viral infections, which modulate innate immune responses and exert positive and negative effects on TLR-dependent responses (53).

Platelets also recognize viral particles coated with immunoglobulins via their FcγRII receptor, which results in Fc receptor-mediated platelet activation, aggregation, and platelet clearance (54, 55). FcγRII-mediated platelet activation depends on IgG and GPIIb/IIIa engagement and involves ADP and thromboxane A_2_ (TxA_2_) feedback mechanisms to cause platelet aggregation, which is further enhanced by CXCL4 (56).

Furthermore, B-lymphocyte production of antibodies against some viruses has been shown to interfere with platelet survival. These antibodies, which usually target surface glycoproteins of viruses, show a cross-reactivity with platelet surface intergrins such as GPIIb/IIIa or GPIb-IX-V (38). This so called idiopathic thrombocytopenic purpura (ITP) or platelet autoantibody-induced thrombocytopenia has been described for HCV, HIV, CMV, EBV, hantavirus, varicella zoster virus, herpes viruses, and severe acute respiratory syndrome coronavirus (38).

Additionally, platelet destruction in response to viral infections can occur due to disturbed portal vein pressure and enhanced sequestration of platelets by the enlarged spleen as is the case in HCV infection (57).

## Multifactorial Mechanisms Leading to Thrombocytopenia

Thrombocytopenia in response to viral infections is often multifactorial. In viral hepatitis, thrombocytopenia is caused by platelet-specific glycoprotein antibodies (58) as well as by immune complexes bound to the platelet surface (59). In the case of HCV, thrombocytopenia can be reversed using a selective thrombin receptor agonist (60), indicating that coagulation, inflammation, and platelet activation play a role in HCV-induced decrease of platelet count. HCV also indirectly affects megakaryopoiesis (25, 59). HCV-induced liver dysfunction results in a decrease in TPO production, which in turn results in impaired platelet production in the bone marrow (20). The recovery of TPO levels and increase in platelet count following successful liver transplantations in HCV-infected individuals (20) emphasizes the importance of this mechanism in HCV-induced thrombocytopenia. Moreover, portal hypertension leads to platelet activation and shortens platelet survival due to increased splenic platelet sequestration (57). However, treatment aiming at reversing portal hypertension did not always correct thrombocytopenia (20).

Human immunodeficiency virus-induced thrombocytopenia is caused by multiple mechanisms. The effects of HIV on megakaryocyte development were recently reviewed in Ref. (30). In brief, HIV results in impaired survival of bone marrow megakaryocytes and their precursors. HIV also decreases the number and activity of human progenitor cells and decreases megakaryocyte maturation and ploidy. HIV surface glycoprotein gp120 leads to increased megakaryocyte apoptosis *in vitro* due to increased TGFβ and down-regulation of the proliferation-inducing ligand tumor necrosis factor ligand superfamily member 13 (TNFSF13). Further, gp120 interacts with CD4, which is expressed by immature megakaryocytes, which also express CCR5, and leads to their infection (61). Furthermore, HIV infection of megakaryocytes can lead to reduced TPO receptor (c-Mpl) expression.

In dengue virus infection, platelet production is impaired by suppression of megakaryopoiesis via infection of hematopoietic progenitor cells or indirectly via altered cytokine levels in the bone marrow due to impaired stromal cell function (51). Platelets from patients with dengue infection present signs of activation, mitochondrial dysfunction, and enhanced apoptosis, which may contribute to the genesis of thrombocytopenia (62, 63). Further, enhanced destruction of platelets occurs due to cross-reaction of platelets with anti-dengue virus antibodies. Dengue virus-induced anti-non-structural protein-1 (NS-1) induces complement-mediated lysis of platelets and thereby further accelerates thrombocytopenia (64). NS-1 can also activate endothelial cells and leads to increased vascular permeability and further platelet activation (65). Dengue virus-infected patients show increased levels of E-selectin on their endothelial cell surface, which promotes adhesion and clearance of platelets (65, 66) as well as enhanced activation of the coagulation cascade (67).

Arenaviruses infection by either lymphocytic choriomeningitis virus (LCMV) or Junin virus results in thrombocytopenia and decreased agonist-induced platelet responses in mice (68, 69). As a consequence, platelet depletion in LMCV-infected mice results in lethal hemorrhagic anemia (68). This effect is caused by diminished platelet responses, rather than solely a drop in platelet count. The underlying mechanism of altered platelet production and reduced platelet reactivity was found to rely on virus-induced production of interferon (IFN) α/β (68, 69). Junin virus mainly infects CD34^+^ cells not megakaryocytes but impairs proplatelet formation and platelet release via IFNα/β receptor signaling (69). IFNα/β receptor signaling represents an important paracrine repressor of megakaryopoiesis, which directly inhibits TPO-induced signaling through induction of suppressor of cytokine signaling 1 (SOCS-1) (70), induction of 2′5′-oligoadenylate synthetase (OAS) (68), and decease of nuclear factor erythroid 2 (NF-E2) expression (69).

Hantavirus can directly interact with and activate platelets via GPIIb/IIIa (35) and infection of megakaryocytes with hantavirus induces the up-regulation of human leukocyte antigen (HLA) class 1 molecules on the megakaryocyte surface, which leads to cytotoxic T-lymphocyte-mediated destruction of megakaryocytes (27).

Viruses also possess enzymes, which can modulate platelet functions. For example, Influenza virus exhibits neuraminidase (sialidase), which hydrolyses the terminal sialic acid residues from host cell receptors and thereby decreases the life span of platelets by targeting platelets for rapid clearance in the liver and spleen (71). As another example, mycoviral neuraminidase has been shown to reduce platelet life span by cleaving sialic acid in the platelet membrane (72). Besides the effect on platelet live span, neuroaminidase further alters megakaryocyte ploidy as well as morphology and size of platelets (73). Newcastle virus can directly disrupt platelet cell membrane, resulting in platelet lysis (74). Human parvovirus 19 cannot reproduce in megakaryocytes, but triggers a drop in platelet count via platelet activation (75).

Despite its effects on TPO generation, direct interaction of the non-human SIV with platelets results in platelet–monocyte aggregate formation, which promotes monocyte differentiation into a more inflammatory phenotype. Furthermore, platelet activation triggers clearance of platelets (76, 77) as platelets become recognized by macrophages in the spleen, leading to a rapid drop in platelet count.

It is currently unclear if, and to what extent, viruses or the host could benefit from thrombocytopenia. It has been suggested that down-regulation of hematopoiesis, for example, in dengue virus infection might have a protective role for the microenvironment to limit injury during elimination of infected cells (78).

## Antiviral Effects of Platelets

Direct interaction between platelets and viruses has been observed in various viral infections and could be proven by *in vitro* studies. The interactions leading to platelet activation do not only result in enhanced platelet clearance, the elimination of virus-laden platelets also contributes to the clearance of virus particles (25).

**Figure 3:**
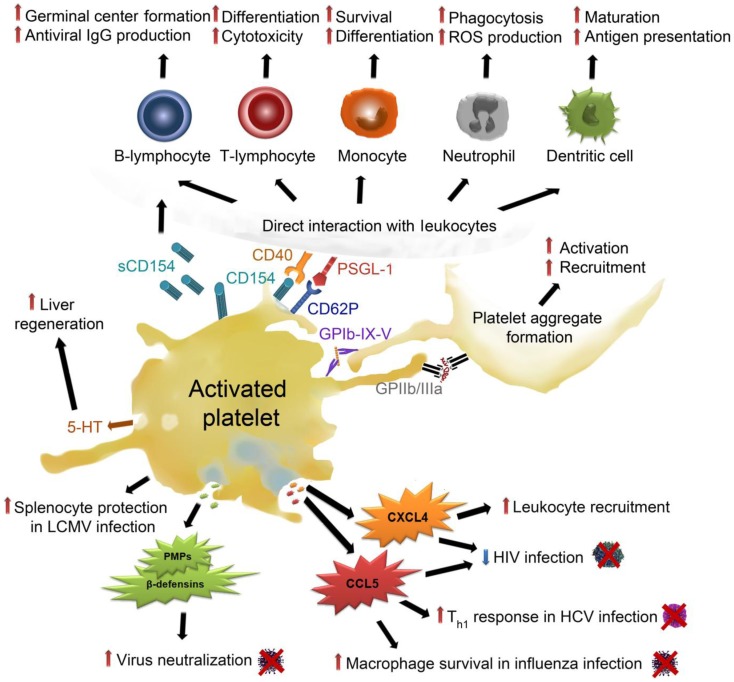
**Effects of activated platelets on virus infection: upon activation, platelets release their α-granules, containing high amounts of CXCL4, which up-regulates coagulation and leukocyte recruitment**. Moreover, CXCL4 decreases HIV infection but also enhances liver fibrosis. CCL5, another α-granule-derived chemokine, also decreases HIV infection and enhances T_h1_ lymphocyte responses in HCV infection and serves as a survival signal for macrophages in influenza infection. Platelets release PMPs and β-defensins from their α-granules, which mediate virus neutralization. They protect splenocytes from necrosis in LCMV infection. Platelet dense granules contain 5-HT, which enables liver regeneration but also HBV and HCV infection. Platelet activation leads to the expression and release of CD154 and CD62P, which allow direct interaction between platelets and leukocytes. Platelet interaction with B-lymphocytes enhances germinal center formation and antiviral IgG production. Platelets trigger T-lymphocyte differentiation and cytotoxicity as well as survival and differentiation of monocytes. In neutrophils, platelet adhesion stimulates ROS production and boosts phagocytosis. Platelet interaction with dendritic cells promotes their maturation and facilitates antigen presentation. Finally, platelet activation results in interaction and activation of further platelets, which triggers platelet aggregation and amplifies the above described processes. CCL5, chemokine (C–C motif) ligand 5; CXCL4, C–X–C chemokine ligand 4, GP, glycoprotein; HBV, hepatitis B virus; HCV, hepatitis C virus; HIV, human immunodeficient virus; IgG, immunglobulin G; LCMV, lymphocytic choriomeningitis; PMPs, Platelet antimicrobial peptides; PSGL-1, P-selectin (CD62P) glycoprotein ligand-1; sCD154, soluble CD154/CD40 ligand; Th1, T-helper lymphocyte type 1; 5-HT, serotonin.

An overview on the different mechanisms by which platelets can interfere with virus infection is given in Figure 3. Platelet activation in response to direct and indirect interaction with viruses results in platelet activation and degranulation. Platelet α-granules provide platelet-mediated host defense mechanisms, as they contain kinocidins and microbicidal proteins important for antiviral host defense. Kinocidins have immune-modulatory functions including chemotaxis and activation of various immune cells (79). They can differentiate between host and pathogen cell membranes and distinct microbial spectra under different pH conditions. Thrombin, which is produced upon inflammation and injury, can proteolytically cleave kinocidins and platelet antimicrobial peptides resulting in cleavage products with a stronger and broader range of antimicrobial activity (49, 80). Pathogens can also cleave kinocidins on distinct sites, which potentially results in their inactivation.

The most abundant platelet kinocidin is platelet factor 4 (PF-4/CXCL4). CXCL4 is released into the blood stream upon tissue injury, inflammation, oxidative stress, or pathogen stimulation such as platelet–virus interactions. CXCL4 has been identified as a broad-spectrum HIV-1 inhibitor and suppresses HIV-1 infection of T-lymphocytes via steric inhibition by binding of CXCL4 proximal to the CD4 binding site on gb120 (81, 82).

Other important chemokine released during viral infections in host defense are CCL5 and CCL3 (83). CCL5 and CCL3 have been identified as major HIV-suppressive factors produced by cytotoxic T-lymphocytes (84) and also platelet-derived CCL5 and CCL3 could play a role in this process. It is currently unclear whether the effect of CCL5 on inhibiting HIV infection is due to perturbation of the viral envelope and/or due to competitive binding to its receptors (primarily CCR5) (79). HIV gp41 is able to decrease CCL5 release from platelets, thereby directly counteracting the antiviral responses of platelets (85). While CXCL4 is mainly platelet-derived, CCL5 can be released by various cells types and indeed, a variety of viruses has been shown to induce CCL5 release [reviewed in Ref. (86)].

CCL5 is further involved in viral lung diseases (87). It represents an important chemokine during the development and resolution of a pulmonary leukocyte response to influenza A virus infection in mice (88). CCL5 interaction with its receptor CCR5 provides an anti-apoptotic signal for macrophages during influenza virus infection (89) and is important for cell survival during viral infections. CCL5 has multifaceted roles in HCV infection, as it modulates inflammatory reactions and tissue injury and also induces the up-regulation of T-lymphocyte helper cells type 1 (Th1) (90).

Upon activation, platelets release β-defensins, which have been shown to neutralize a variety of viruses (91). Platelet antimicrobial peptides PD3 (KNGRKLCLDLQAALY) and PD4 (AALYKKKIIKKLLES), which are platelet α-granule-derived molecules, have also been demonstrated to potently reduce viral titers of vaccinia virus (92). Other platelet-derived antimicrobial peptides, for example, thymosin beta 4, CXCL7, or cleavage products of antimicrobial peptides (fibrinopeptide A and B, thrombocidins), do not directly act against viral infections but could prevent the host from bacterial super-infections (49).

The highest amounts of serotonin in blood are stored in platelet dense granules, which are also released upon platelet activation. Serotonin was found to be an important mediator of liver regeneration (93, 94) and also mediates early T-lymphocyte stimulation (95).

Platelet degranulation further results in surface expression of P-selectin, which is part of the inner α-granule membrane, which fuses with the outer platelet membrane upon granule exocytosis. P-selectin interacts with its counter-receptor P-selectin glycoprotein ligand-1 (PSGL-1), which is constitutively expressed on the surface of leukocytes (96). Platelet interactions with leukocytes mediate immune responses during viral infections (79). Direct interaction with platelets leads to activation of leukocytes, resulting in enhanced phagocytosis and reactive oxygen production of neutrophils, as well as neutrophil extracellular trap formation. The platelet–monocyte interaction results in an increased activation and differentiation and boosts monocyte surface expression of TF and formation of microparticles (96).

In response to CMV and EMCV, platelet–virus interaction results in stimulation of TLRs, which trigger platelet degranulation and rapid direct interaction of platelets and neutrophils (32, 33). Of note, platelets were shown to decrease EMCV counts in a TLR7-dependent fashion and contribute to host survival while no pro-thrombotic consequences of platelet TLR7 and EMCV interaction could be observed (33).

Platelets recruit dendritic cells to sites of injury or infection (97) and products released from platelets promote maturation of dendritic cells and enhance their antigen presenting capacity (98, 99).

Platelet–leukocyte interaction and modulation of leukocyte functions can be further accelerated by CD40 ligand (CD40L/CD154) binding to CD40. Platelets express and secrete (soluble) CD154, thereby triggering host responses and boosting inflammation.

Platelets directly interact with T-lymphocytes and B-lymphocytes and modulate their function via direct cell–cell interaction as well as soluble mediators (100). Platelet-released CXCL4 and CCL5 enhance pro- and anti-inflammatory cytokine production of T-lymphocytes (101). CXCL4 represents an important mediator in T-cell differentiation, which leads to an increase in regulatory T-lymphocytes (102) and limits Th17 differentiation (103).

Platelets additionally enhance T-lymphocyte-mediated germinal center formation (104) and they can independently up-regulate adenovirus specific IgGs via CD154 (105).

Binding of IgG-coated viruses causes platelet expression of CD154 and CCL5, which in turn primes protective T-cell-mediated immunity (79). Platelets in mice infected with lymphocytic choriomeningitis virus were found to be responsible for efficient cytotoxic T-cell responses, which depended on platelet GPIIIa (CD61) and CD154 (68). T-lymphocyte interaction with platelets also results in the activation of platelets, which amplifies the release of CCL5 (106).

In hepatitis virus B (HBV) infected mice, platelets were shown to be responsible for the intra-hepatic accumulation of virus-specific cytotoxic T-lymphocytes (107) and to mediate cytotoxic T-cell influx into liver tissue via P-selectin, which enhances viral clearance but also tissue damage (108). It was further shown an LCMV infection model that a drop in platelet count leads to necrotic destruction of splenocytes (109). This affects innate and adaptive immunity and leads to uncontrolled LCMV replication (109), indicating that platelets are necessary to maintain splenocyte survival.

## Adverse Effects of Platelet Activation in Viral Infections

**Figure 4:**
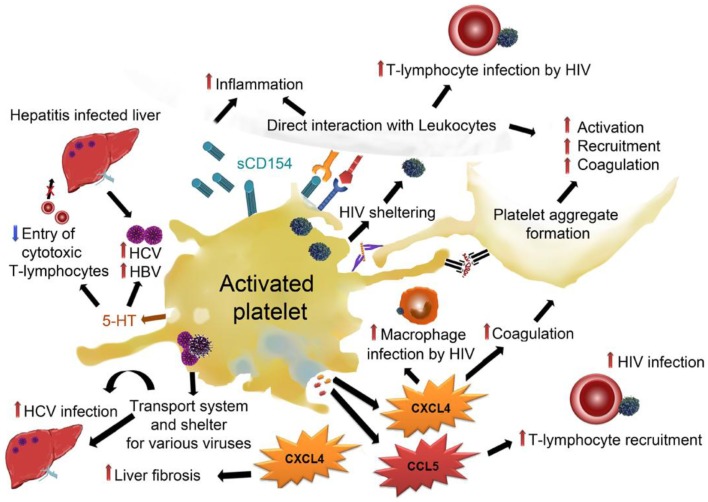
**Adverse effects of platelets in viral infections: platelets can shelter viruses like HIV, HVC, HBV, and influenza and facilitate their transportation throughout the circulation, thereby enabling *de novo* infections at distal sites**. Platelet–virus interaction often results in platelet activation and subsequent release of α-granules and dense granules. CXCL4 from α-granules has been demonstrated to enhance HIV infection of macrophages and enhance liver fibrosis in hepatitis. CCL5 enhances T-lymphocyte recruitment and thereby promotes HIV infection of T-lymphocytes. Dense granule-derived 5-HT was shown to boost HBV and HCV infection by decreasing the entry of cytotoxic T-lymphocytes. Direct interaction of platelets with leukocytes facilitates infection of leukocytes and enhances both leukocyte activation and inflammation. Activation of platelets leads to activation and recruitment of further platelets and enhances pro-coagulatory processes.CCL5, chemokine (C–C motif) ligand 5; CXCL4, C–X–C chemokine ligand 4, GP, glycoprotein; HBV, hepatitis B virus; HCV, hepatitis C virus; HIV, human immunodeficient virus; sCD154, soluble CD154/CD40 ligand; 5-HT, serotonin.

To date, it remains unclear if platelet–virus interactions are beneficial for the host or for the virus. Potential adverse effects of platelets in viral infections are summarized in Figure 4. HIV, for example, is internalized by platelets, which results in sheltering of virus particles from the host immune systems and allows dissemination throughout the entire body [reviewed in Ref. (25)]. Moreover, in response to HIV interaction, platelets become activated and release CCL5, which results in the recruitment of highly susceptible target cells like T-lymphocytes and monocytes. It was demonstrated for influenza virus that platelets serve as a carrier for the virus in the circulation (72). Also HCV has been shown to take advantage of platelets as a safe transport system to reach the liver, where platelet activation further enhances the interaction of platelets and liver cells. This interaction prolongs the time for potential infection of liver tissue by the virus (46).

Virus-mediated activation of platelets and subsequent platelet-derived cytokine release not only protects the host but may also have unwanted implications for the host. While several reports indicate that CXCL4 is a broad-spectrum suppressor of HIV infection, one report indicates that platelet CXCL4 can also facilitate human macrophage infection with HIV-1 and potentiates virus replication (110). Furthermore, CXCL4 has been shown to mediate liver fibrosis in experimental mouse models (111), indicating that this chemokine could play a role in platelet-mediated acceleration of hepatitis-induced liver fibrosis.

Platelet-derived serotonin results in a delayed entry of activated cytotoxic T-lymphocytes into the liver, which slows down virus control and supports virus persistence in the liver (112). This results in aggravation of virus-induced immunopathology.

Megakaryocyte infection and subsequent modulation of platelet function by CMV have been speculated to be a reason for graft failure in allergenic bone marrow transplantation (25).

## Bleeding and Thrombotic Complications in Response to Viral Infection

Primary and secondary hemostasis work tightly together. Imbalances of either system result in impaired function of the other. Activation of the coagulation cascade has been observed in various virus infections, including HIV, dengue, and Ebola virus infection, and might provide a host defense mechanism to limit pathogen dissemination (53). Changes in the activation of the coagulation cascade and modulation of platelet count and function, which are also observed during viral infections, lead to an increased risk of disseminated vascular coagulation (DIC), deep vein thrombosis (DVT) thrombosis, and hemorrhage in infected patients (38). Thrombocytopenia is a common result of viral infections and associated with an increased bleeding risk. Approximately 10% of HIV positive patients and up to 60% of patients with acquired immunodeficiency syndrome (AIDS) suffer from thrombocytopenia, which can lead to severe bleedings in these patients (30). In many viral infections, platelet function and aggregation in response to different agonists are diminished (25), causing bleeding complications in viral hemorrhagic fevers (VHF) (51). VHF outbreaks lead to the deaths of thousands of people every year and are caused by different enveloped RNA viruses, which include *Arenaviridae* (e.g., Lassa virus), *Bunyaviridea* (e.g., hantavirus), *Filioviridae* (e.g., Marburg and Ebola virus), and *Flaviviridae* (e.g., dengue virus).

Of note, it was recently shown that a reduction in platelet count of more than 85% is necessary for hemorrhages to occur, indicating that a very low percentage of platelets are sufficient to maintain vascular integrity (109). In an LCMV model, it was demonstrated that even severe thrombocytopenic mice develop only local hemorrhages at sites of inflammation and that LCMV-dependent bleedings are a result of IFNα/β signaling-induced platelet dysfunction (68). Mice lacking functional IFNα/β receptor show less severe anemia and hemorrhages due to restored platelet aggregation capacity during LCMV infection (68). This indicates that platelet dysfunction has more tremendous effects than thrombocytopenia in these pathologies. Of note, many VHF viruses inhibit platelet function. Junin virus, causative of Argentinean hemorrhagic fever, induces IFNα/β signaling-dependent decrease in platelet production and function (69). Ebola virus also induces an increase in IFNα, which correlated with increased fatality (113). Ebola infection further triggers TF expression, which is associated with Ebola hemorrhagic fever (114). Hantavirus and Lassavirus also abrogate platelet responses via plasma-mediated platelet inhibition and/or direct interaction (37, 115).

Although thrombocytopenia is frequently observed in patients with dengue infections, bleedings are rare. However, if bleedings do occur they are associated with high mortality risk. Of note, in dengue virus-infected patients, platelet count does not predict bleeding risk. However, systemic platelet activation might contribute to the pro-coagulatory state in these patients, which frequently develop DIC (67). Moreover, elevated platelet activation is associated with plasma leakage (116). H1N1 influenza infection enhances activation of circulating platelets (117) and results in increased events of thrombosis (118).

In line with the observation that platelet dysfunction increases the risk of hemorrhages and therefore mortality, it has been demonstrated that pharmacological inhibition of either platelets or the coagulation cascade increases the mortality of H1N1-infected mice (53). Thus, aspirin treatment, which inhibits platelet activation via inhibition of cyclooxygenase and subsequent TxA_2_ production, has been hypothesized to have worsened the incidence and severity of the influenza pandemics in the 1910s (119).

Taken together, viruses can either enhance platelet activation resulting in pro-thrombotic events or diminish platelet responses thereby leading to bleeding complications. Platelet count alone does not seem to be sufficient to predict adverse platelet effects and parameters like platelet activation and reactivity might be more accurate to predict thrombotic or bleeding risks in patients.

## Platelet–Virus Interplay in Cardiovascular Disease

Platelets are responsible for the lethal consequences of cardiovascular disease but emerging evidence suggests that they also play a role in the initiation and progression of atherosclerosis. Several viruses have been associated with cardiovascular disease and herpes simplex virus (HSV), HIV, CMV, HCV, EBV as well as influenza DNA, and/or protein has been found in atherosclerotic plaques (120, 121). Large cohort studies indicate that seropositivity for CMV or HCV represent an independent risk marker for cardiovascular diseases (122, 123), while other studies could not confirm this observation (121). CMV and HCV are further associated with increased graft rejection rates, restenosis following coronary angioplasty and transplant vascular sclerosis, indicating that latent virus re-activates during immunosuppression and contributes to adverse effects (121).

In animal studies, CMV increased T-lymphocyte influx into plaques (124) and neutrophil extravasation, which is further supported in the presence of platelets (32). Influenza virus infection correlates with acute coronary syndromes and myocardial infarction (125), while influenza seropositivity and cardiovascular disease show no clear correlation (121). Animal experiments revealed that influenza triggers inflammatory and thrombotic responses in atherosclerotic plaques (126) and reverses the protective role of high-density lipoproteins (127). Both mechanisms are likely to modulate platelet function and reactivity.

There is some discrepancy regarding the strength of data regarding HSV infection and cardiovascular disease and despite its pro-inflammatory responses, a negative association between EBV infection and cardiovascular disease has been observed.

Moreover, the association between HIV infection and cardiovascular disease remains controversial and anti-retroviral therapy itself alters platelet function (128) and represents an independent risk factor for atherosclerosis (121). Further studies are warranted to evaluate the contribution of platelet–virus interactions in cardiovascular disease.

## Conclusion

Platelets and their released products have been reported to directly and indirectly suppress infection but also to support virus persistence in response to certain viruses, making platelets a double-edged sword during viral infections. Platelets are involved in a variety of complications in response to viral infection but also fulfill a pivotal role in retaining adequate host responses. Thrombocytopenia is a common complication in several viral infections and viruses exert various strategies to mediate platelet decay. The question, if thrombocytopenia is a viral strategy to evade immune responses or if it also has host protecting functions, seems to depend on the virus variant and the underlying pathology. Further studies are warranted to fully understand the role of platelets in viral infections and to gain a clear understanding of the effects of anti-platelet therapies in viral infections. These studies will help us to predict the benefit or drawback of platelets and their inhibition during viral infections.

## Conflict of Interest Statement

The author declares that the research was conducted in the absence of any commercial or financial relationships that could be construed as a potential conflict of interest.
